# (*E*)-4-{[(4-Meth­oxy­naphthalen-1-yl)methyl­idene]amino}­phenol

**DOI:** 10.1107/S2414314626008552

**Published:** 2026-08-28

**Authors:** Amel Ghames, Nouara Ziani, Amel Djedouani, Douniazed Hannachi, Aurelian Crochet, Helen Stoeckli-Evans

**Affiliations:** a Laboratoire d’électrochimie des matériaux moléculaires et des complexes, (LEMMC), Département de génie des procédés, Université Ferhat Abbas, Sétif 19000, Algeria; b Département de chimie Faculté des sciences Université Ferhat Abbas, Sétif 19000 Algeria; c Ecole Normale Supérieure de Constantine-Assia Djebar, Université Constantine 3, 25000, Ali Mendjli, Algeria; d Laboratoire des Produits Naturels d’Origine Végétale et de Synthése Organique, Faculté des Sciences Exactes, Université des Fréres Mentouri-Constantine 1, Algeria; e Laboratoire d’électrochimie, d’ingénierie moléculaire et de catalyse redox, Département de génie des procédés, Faculté de technologie, Universite Ferhat Abbas, Sétif 19000, Algeria; f Chemistry Department, University of Fribourg, Chemin du Musée 9, CH-1700 Fribourg, Switzerland; g Institute of Physics, University of Neuchâtel, Rue Emile-Argand 11, CH-2000 Neuchâtel, Switzerland; University of Aberdeen, United Kingdom

**Keywords:** crystal structure, naphthalene, Schiff base, hydrogen bonding, C—H⋯π inter­actions

## Abstract

The title Schiff base compound, C_18_H_15_NO_2_, was synthesized *via* the condensation reaction of 4-hy­droxy aniline and 4-meth­oxy-1-naphthaldehyde in ethanol. The dihedral angle between the phenol ring and the mean plane of the naphthalene ring system is 75.81 (6)°. In the crystal, the mol­ecules are linked by O—H⋯N and C—H⋯O hydrogen bonds, enclosing *R*^3^_3_(18) ring motifs and forming zigzag chains propagating along the *c*-axis direction. The chains are linked *via* C—H⋯π inter­actions forming slabs lying parallel to the *ac* plane.

## Abstract 2

The title compound was obtained from the reaction of 4-hy­droxy-aniline and 4-meth­oxy-1-naphthaldehyde in ethanol. The dihedral angle between the phenol ring and the mean plane of the naphthalene ring system is 75.81 (6)°.

## Graphical abstract

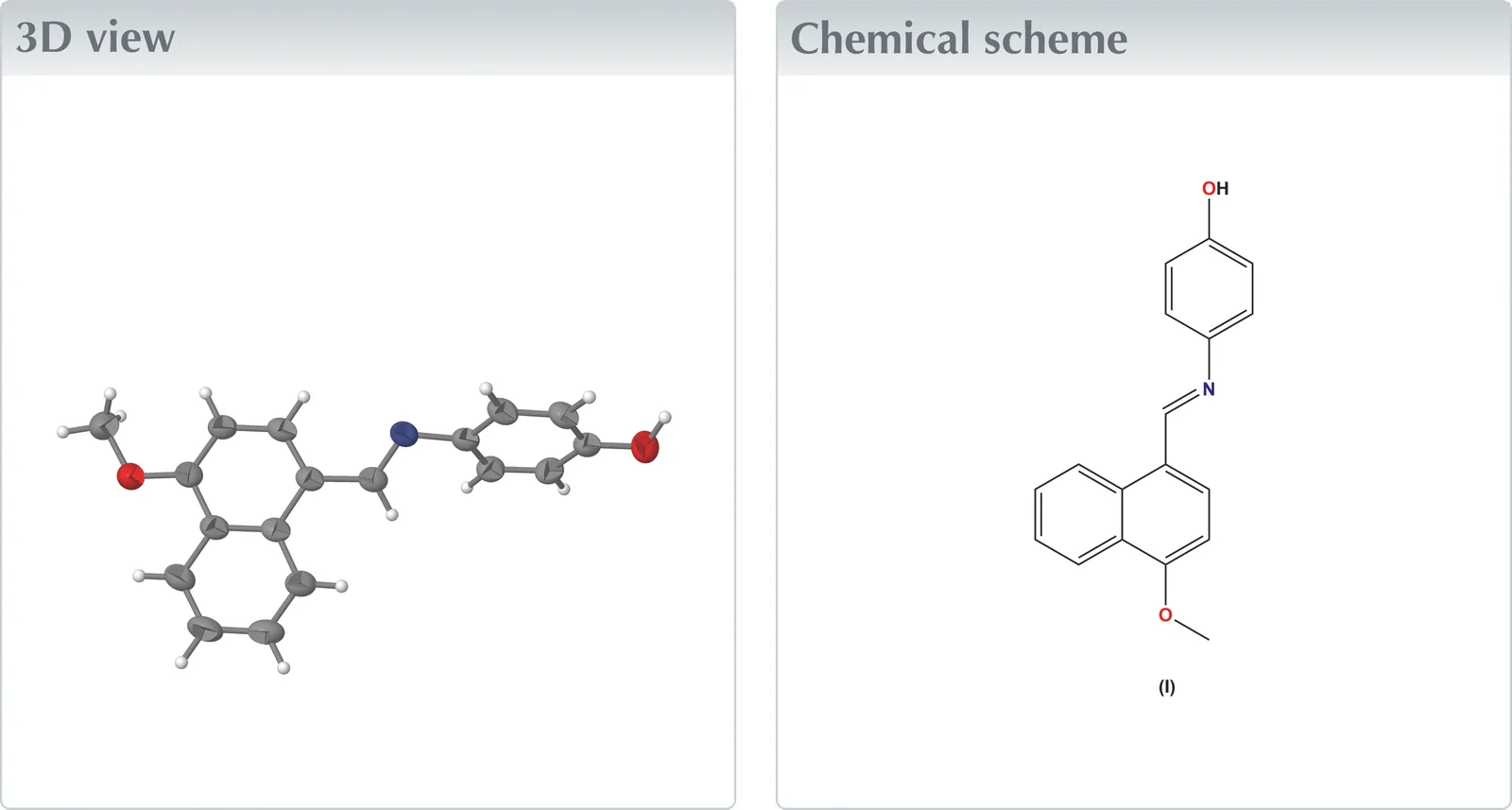

## Structure description

Schiff bases exhibit a wide range of biological activities (Djabbour *et al.*, 2025 ▸; Villar *et al.*, 2004 ▸). They have applications in the field of water treatment as they have a great capacity for complexation of transition metals (Boudraa *et al.*, 2023 ▸; Kalcher *et al.*, 1995 ▸). Their use as corrosion inhibitors (*e.g.*, Boudraa *et al.*, 2023 ▸) reveal their importance in this field too. Herein, we report on the synthesis and crystal structure of the title compound, C_18_H_15_NO_2_ (**I**).

**Figure 1:**
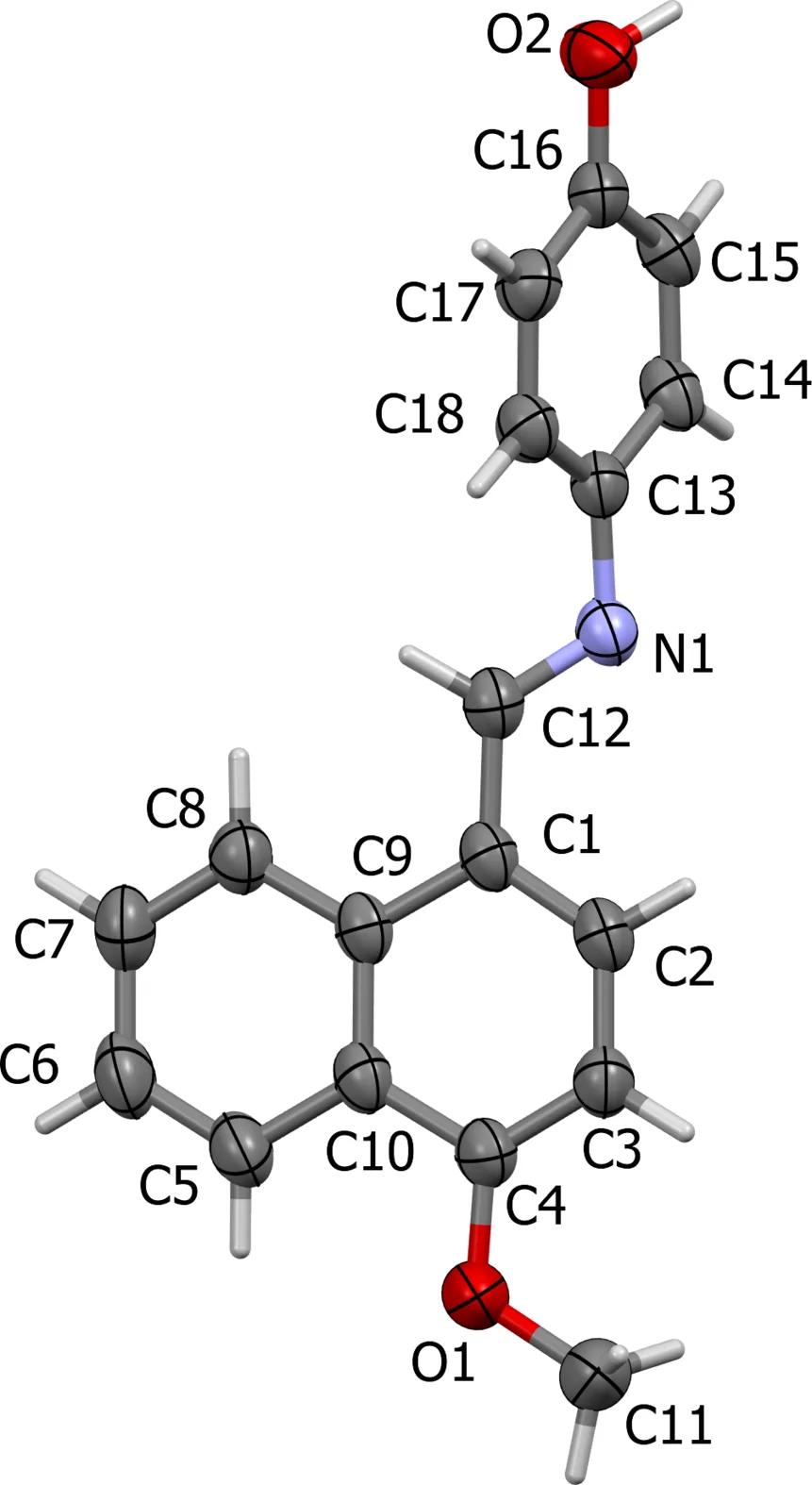
A view of the mol­ecular structure of compound (**I**). The displacement ellipsoids are drawn at the 50% probability level.

The mol­ecular structure of compound (**I**) is illustrated in Fig. 1 ▸. The configuration about the N1=C12 azomethine bond is *E*. The –C=N– bond length [1.282 (2) Å] is a little longer than the bond lengths observed in related compounds, such as (*E*)-1-(4-meth­oxy­naphthalen-1-yl)-*N*-phenyl­methanimine (**II**) [1.264 (4) Å; Linden *et al.*, 1989 ▸] and (*E*)-2-{[(4-meth­oxy­naphthalen-1-yl)methyl­ene]amino}-4-methyl­phenol (**III**) [1.274 (2) Å; Yahiaoui *et al.*, 2023 ▸]. A search of the Cambridge Structural Database (CSD, Version 6.01; last update May 2026; Groom *et al.*, 2016 ▸) for the length of the azomethine bond of structures with the benzyl­ideneaniline substructure gave 1601 hits (Filters: *R* ≤ 0.05, no disorder, no ions, no polymers, single crystals and organics only). An analysis using *Mercury* (Macrae *et al.*, 2020 ▸) gave an average value for the –C=N– bond length of 1.277 (15) Å, with a median value of 1.278 Å. Hence, the N1=C12 bond length in (**I**) is normal.

The dihedral angle between the phenol ring (C13–C18) and the mean plane of the naphthalene ring system (C1–C10; r.m.s. deviation 0.013 Å) in (**I**) is 75.81 (6)°. In compound (**II**) the corresponding dihedral angle is 64.33 (12)°, while in compound (**III**) the same dihedral angle is smaller, at 33.41 (4)° probably due to the presence of an intra­molecular O—H⋯N hydrogen bond.

**Figure 2:**
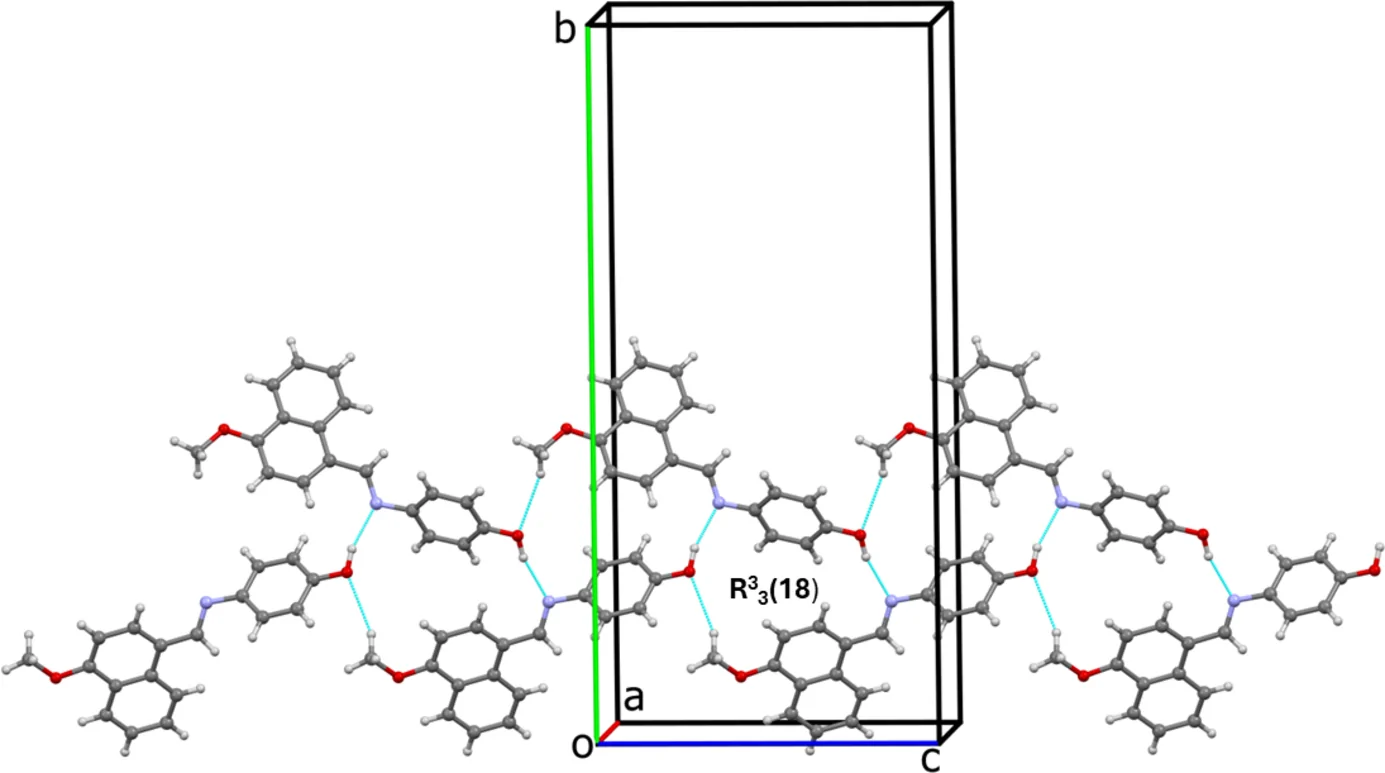
A partial view along the *a*-axis of the crystal structure of compound **I**, illustrating the formation of the zigzag chain *via* O—H⋯N and C—H⋯O hydrogen bonds and the formation of the 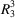
(18) ring motifs (Table 1 ▸). The various hydrogen bonds are shown as cyan dashed lines.

**Figure 3:**
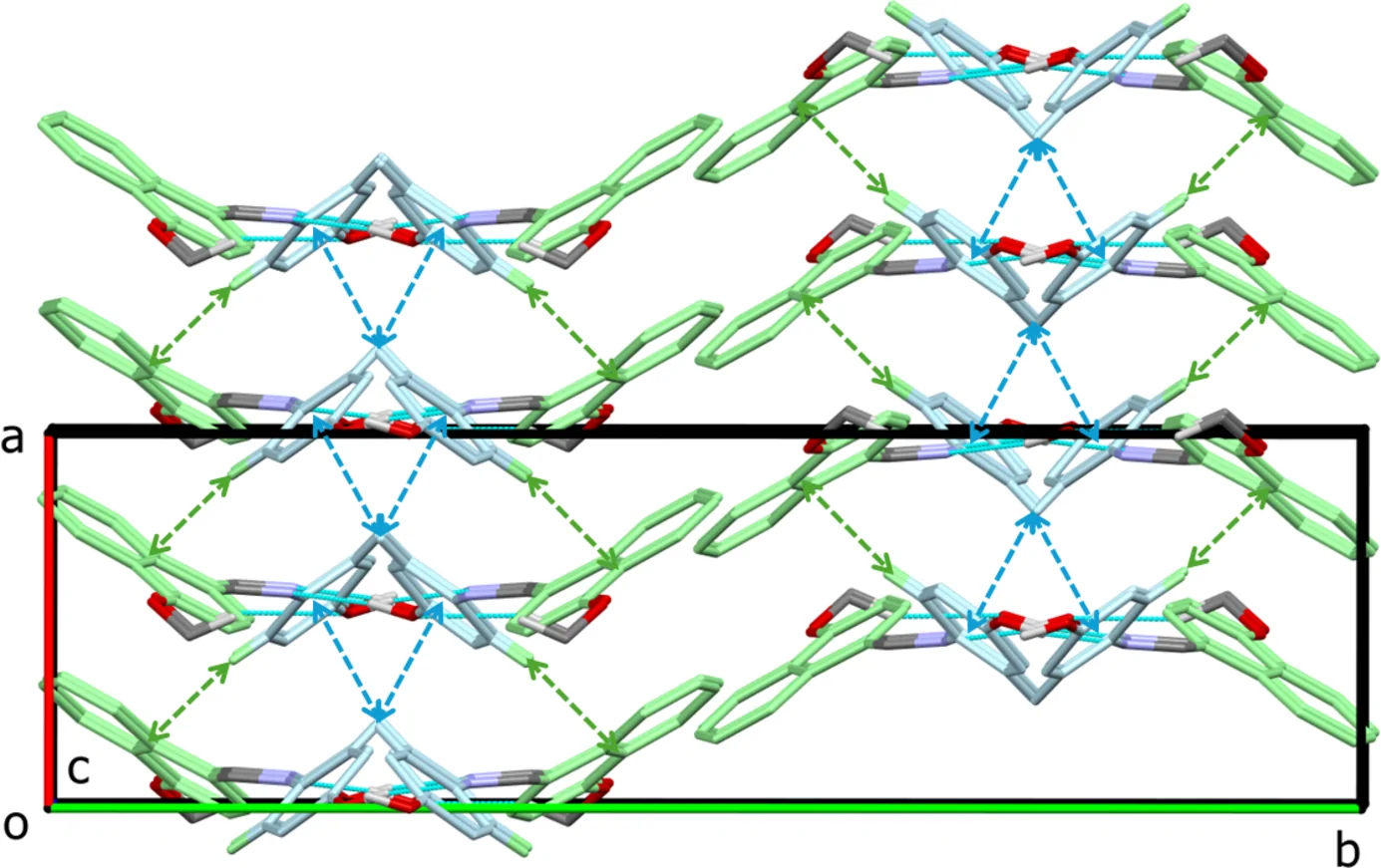
A view along the *c*-axis of the crystal packing of compound **I**. The C—H⋯π inter­actions are shown as dashed double arrows (see Table 1 ▸; naphthalene ring system is pale green and the phenol ring is pale blue).

In the crystal of (**I**), the mol­ecules are linked by O—H⋯N hydrogen bonds to form zigzag chains propagating along the *c*-axis direction (Fig. 2 ▸). The chains are consolidated by C—H⋯O hydrogen bonds, so enclosing 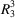
(18) ring motifs (Fig. 2 ▸, Table 1 ▸). The chains are linked by C—H⋯π inter­actions forming slabs lying parallel to the *ac* plane (Fig. 3 ▸, Table 1 ▸). The slabs stack along the *b*-axis direction and there are no significant inter-planar inter­actions present.

## Synthesis and crystallization

Equimolar amounts of 4-hy­droxy aniline (0.109 g, 0.01 mmol) and 4-meth­oxy naphthaldehyde (0.186 g, 0.01 mmol) were mixed in absolute ethanol (30 ml) and refluxed under stirring for 4 h. After cooling to room temperature, the solvent was allowed to evaporate slowly, yielding yellow needle-like crystals of (**I**).

## Refinement

Crystal data, data collection and structure refinement details are summarized in Table 2 ▸.

## Supplementary Material

Crystal structure: contains datablock(s) I. DOI: 10.1107/S2414314626008552/hb4570sup1.cif

Structure factors: contains datablock(s) I. DOI: 10.1107/S2414314626008552/hb4570Isup2.hkl

Supporting information file. DOI: 10.1107/S2414314626008552/hb4570Isup3.cml

CCDC reference: 2582009

Additional supporting information:  crystallographic information; 3D view; checkCIF report

## Figures and Tables

**Table 1:** Hydrogen-bond geometry (Å, °) *Cg*1 and *Cg*2 are the centroids of the naphthalene ring system (C1–C10) and the phenol ring (C13–C18), respectively.

*D*—H⋯*A*	*D*—H	H⋯*A*	*D*⋯*A*	*D*—H⋯*A*
O2—H1⋯N1^i^	0.91 (3)	1.97 (3)	2.849 (2)	160 (3)
C11—H11*A*⋯O2^ii^	0.97	2.53	3.409 (2)	150
C14—H14⋯*Cg*2^iii^	0.94	2.90	3.627 (2)	135
C18—H18⋯*Cg*1^iv^	0.94	2.82	3.745 (2)	170

Symmetry codes: (i) 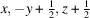; (ii) 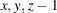; (iii) 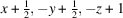; (iv) 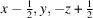.

**Table 2:** Experimental details

Crystal data	
Chemical formula	C_18_H_15_NO_2_
*M*_r_	277.31
Crystal system, space group	Orthorhombic, *Pbca*
Temperature (K)	250
*a*, *b*, *c* (Å)	7.8365 (3), 27.460 (1), 13.0741 (5)
*V* (Å^3^)	2813.43 (18)
*Z*	8
Radiation type	Cu *K*α
μ (mm^−1^)	0.69
Crystal size (mm)	0.54 × 0.24 × 0.09

Data collection	
Diffractometer	Stoe Stadivari
Absorption correction	Analytical (*X-RED32* and *LANA*; Stoe, 2025 ▸)
*T*_min_, *T*_max_	0.682, 0.938
No. of measured, independent and observed [*I* > 2σ(*I*)] reflections	17634, 2477, 2243
*R*_int_	0.057
(sin θ/λ)_max_ (Å^−1^)	0.596

Refinement	
*R*[*F*^2^ > 2σ(*F*^2^)], *wR*(*F*^2^), *S*	0.046, 0.131, 1.01
No. of reflections	2477
No. of parameters	195
H-atom treatment	H atoms treated by a mixture of independent and constrained refinement
Δρ_max_, Δρ_min_ (e Å^−3^)	0.19, −0.25

Computer programs: *X-AREAPilatus3_SV*, *X-AREA* and *LANA* (Stoe, 2025 ▸), *SHELXT2019/3* (Sheldrick, 2015*a* ▸), *Mercury* (Macrae *et al.*, 2020 ▸), *SHELXL2019/3* (Sheldrick, 2015*b* ▸), *PLATON* (Spek, 2020 ▸) and *publCIF* (Westrip, 2010 ▸).

## full crystallographic data

### (E)-4-{[(4-Methoxynaphthalen-1-yl)methylidene]amino}phenol Crystal data

**Table:**

C_18_H_15_NO_2_	*D*_x_ = 1.309 Mg m^−^^3^
*M**_r_* = 277.31	Cu *K*α radiation, λ = 1.54186 Å
Orthorhombic, *P**b**c**a*	Cell parameters from 30029 reflections
*a* = 7.8365 (3) Å	θ = 4.7–67.1°
*b* = 27.460 (1) Å	µ = 0.69 mm^−^^1^
*c* = 13.0741 (5) Å	*T* = 250 K
*V* = 2813.43 (18) Å^3^	Needle, yellow
*Z* = 8	0.54 × 0.24 × 0.09 mm
*F*(000) = 1168	

### (E)-4-{[(4-Methoxynaphthalen-1-yl)methylidene]amino}phenol Data collection

**Table:**

Stoe Stadivari diffractometer	2477 independent reflections
Radiation source: Primux 100 micro	2243 reflections with *I* > 2σ(*I*)
Graded multilayer mirror monochromator	*R*_int_ = 0.057
Detector resolution: 5.81 pixels mm^-1^	θ_max_ = 66.9°, θ_min_ = 4.7°
rotation method, ω scans	*h* = −3→9
Absorption correction: analytical (X-Red32 and *LANA*; Stoe, 2025)	*k* = −32→27
*T*_min_ = 0.682, *T*_max_ = 0.938	*l* = −14→15
17634 measured reflections	

### (E)-4-{[(4-Methoxynaphthalen-1-yl)methylidene]amino}phenol Refinement

**Table:**

Refinement on *F*^2^	Primary atom site location: dual
Least-squares matrix: full	Secondary atom site location: difference Fourier map
*R*[*F*^2^ > 2σ(*F*^2^)] = 0.046	Hydrogen site location: mixed
*wR*(*F*^2^) = 0.131	H atoms treated by a mixture of independent and constrained refinement
*S* = 1.01	*w* = 1/[σ^2^(*F*_o_^2^) + (0.0836*P*)^2^ + 0.6531*P*] where *P* = (*F*_o_^2^ + 2*F*_c_^2^)/3
2477 reflections	(Δ/σ)_max_ = 0.004
195 parameters	Δρ_max_ = 0.19 e Å^−^^3^
0 restraints	Δρ_min_ = −0.25 e Å^−^^3^

### (E)-4-{[(4-Methoxynaphthalen-1-yl)methylidene]amino}phenol Special details

**Table:**

Geometry. All esds (except the esd in the dihedral angle between two l.s. planes) are estimated using the full covariance matrix. The cell esds are taken into account individually in the estimation of esds in distances, angles and torsion angles; correlations between esds in cell parameters are only used when they are defined by crystal symmetry. An approximate (isotropic) treatment of cell esds is used for estimating esds involving l.s. planes.
Refinement. The hydroxyl H atom (H1) was located in a difference Fourier map and freely refined. The C-bound hydrogen atoms were placed geometrically (C—H = 0.94–0.97 Å) and allowed to ride on their parent atoms with *U*_iso_(H) = 1.5*U*_eq_(*C*-methyl) and 1.2*U*_eq_(C) for other H atoms.

### (E)-4-{[(4-Methoxynaphthalen-1-yl)methylidene]amino}phenol Fractional atomic coordinates and isotropic or equivalent isotropic displacement parameters (Å^2^)

**Table:**

	*x*	*y*	*z*	*U*_iso_*/*U*_eq_	
O1	0.54483 (17)	0.42260 (4)	−0.11052 (8)	0.0607 (3)	
O2	0.50176 (18)	0.27526 (4)	0.74559 (9)	0.0631 (4)	
H1	0.535 (4)	0.2440 (11)	0.759 (2)	0.112 (9)*	
N1	0.56304 (15)	0.31870 (4)	0.33044 (9)	0.0431 (3)	
C1	0.57080 (17)	0.37828 (5)	0.19453 (11)	0.0408 (3)	
C2	0.48289 (18)	0.35110 (5)	0.12373 (11)	0.0438 (3)	
H2	0.429306	0.322240	0.145278	0.053*	
C3	0.46995 (19)	0.36462 (5)	0.02107 (11)	0.0456 (3)	
H3	0.408485	0.345058	−0.025015	0.055*	
C4	0.54710 (19)	0.40648 (5)	−0.01231 (11)	0.0448 (4)	
C5	0.7243 (2)	0.47925 (5)	0.02372 (13)	0.0544 (4)	
H5	0.717067	0.488614	−0.045305	0.065*	
C6	0.8157 (2)	0.50699 (6)	0.09119 (14)	0.0611 (5)	
H6	0.871221	0.535300	0.068213	0.073*	
C7	0.8272 (2)	0.49353 (5)	0.19402 (14)	0.0566 (4)	
H7	0.890171	0.512896	0.239741	0.068*	
C8	0.74786 (19)	0.45251 (5)	0.22859 (12)	0.0477 (4)	
H8	0.756775	0.444017	0.298037	0.057*	
C9	0.65200 (17)	0.42243 (4)	0.16155 (11)	0.0404 (3)	
C10	0.64038 (18)	0.43649 (4)	0.05746 (11)	0.0423 (3)	
C11	0.4544 (3)	0.39390 (7)	−0.18289 (14)	0.0765 (6)	
H11C	0.462324	0.408915	−0.249878	0.115*	
H11B	0.335501	0.391672	−0.162765	0.115*	
H11A	0.503581	0.361505	−0.185446	0.115*	
C12	0.57358 (18)	0.36320 (5)	0.30148 (11)	0.0433 (3)	
H12	0.583650	0.387430	0.351919	0.052*	
C13	0.55024 (17)	0.30924 (5)	0.43688 (10)	0.0404 (3)	
C14	0.64259 (18)	0.27075 (5)	0.47837 (11)	0.0457 (4)	
H14	0.712289	0.251745	0.435586	0.055*	
C15	0.63350 (19)	0.26002 (5)	0.58132 (12)	0.0461 (4)	
H15	0.701496	0.234978	0.608536	0.055*	
C16	0.52446 (19)	0.28599 (5)	0.64513 (11)	0.0453 (4)	
C17	0.4292 (2)	0.32384 (5)	0.60349 (11)	0.0486 (4)	
H17	0.353863	0.341496	0.645387	0.058*	
C18	0.44399 (19)	0.33576 (5)	0.50134 (11)	0.0448 (3)	
H18	0.381461	0.362145	0.474999	0.054*	

### (E)-4-{[(4-Methoxynaphthalen-1-yl)methylidene]amino}phenol Atomic displacement parameters (Å^2^)

**Table:**

	*U*^11^	*U*^22^	*U*^33^	*U*^12^	*U*^13^	*U*^23^
O1	0.0941 (9)	0.0422 (6)	0.0456 (6)	−0.0098 (5)	−0.0054 (5)	0.0026 (4)
O2	0.0974 (9)	0.0459 (6)	0.0459 (6)	0.0143 (6)	0.0037 (5)	0.0037 (5)
N1	0.0487 (7)	0.0341 (6)	0.0465 (7)	−0.0005 (5)	0.0025 (5)	0.0007 (5)
C1	0.0435 (7)	0.0305 (6)	0.0484 (8)	0.0030 (5)	0.0021 (5)	0.0000 (5)
C2	0.0469 (7)	0.0312 (6)	0.0534 (8)	−0.0015 (6)	0.0022 (6)	0.0008 (6)
C3	0.0521 (8)	0.0352 (7)	0.0496 (8)	−0.0023 (6)	−0.0043 (6)	−0.0032 (6)
C4	0.0550 (8)	0.0337 (7)	0.0459 (8)	0.0037 (6)	0.0005 (6)	−0.0003 (6)
C5	0.0684 (10)	0.0363 (7)	0.0585 (9)	−0.0048 (7)	0.0056 (7)	0.0038 (6)
C6	0.0711 (11)	0.0369 (8)	0.0752 (11)	−0.0145 (7)	0.0048 (8)	0.0013 (7)
C7	0.0594 (10)	0.0381 (7)	0.0723 (11)	−0.0078 (7)	−0.0045 (7)	−0.0074 (7)
C8	0.0522 (8)	0.0368 (7)	0.0541 (8)	0.0003 (6)	−0.0037 (6)	−0.0028 (6)
C9	0.0418 (7)	0.0293 (6)	0.0501 (8)	0.0045 (5)	0.0020 (6)	−0.0018 (5)
C10	0.0470 (8)	0.0300 (6)	0.0500 (8)	0.0026 (5)	0.0034 (6)	−0.0014 (5)
C11	0.1276 (18)	0.0512 (10)	0.0508 (10)	−0.0136 (10)	−0.0176 (10)	0.0001 (7)
C12	0.0464 (8)	0.0342 (7)	0.0493 (8)	−0.0001 (6)	−0.0001 (6)	−0.0012 (6)
C13	0.0452 (7)	0.0315 (6)	0.0446 (7)	−0.0036 (5)	−0.0014 (5)	−0.0010 (5)
C14	0.0477 (8)	0.0330 (7)	0.0563 (9)	0.0034 (5)	0.0086 (6)	0.0022 (6)
C15	0.0499 (8)	0.0330 (7)	0.0553 (8)	0.0031 (6)	0.0001 (6)	0.0070 (6)
C16	0.0583 (8)	0.0339 (7)	0.0437 (8)	−0.0025 (6)	−0.0039 (6)	−0.0011 (5)
C17	0.0601 (9)	0.0389 (7)	0.0467 (8)	0.0083 (6)	−0.0005 (7)	−0.0064 (6)
C18	0.0519 (8)	0.0346 (7)	0.0480 (8)	0.0077 (6)	−0.0045 (6)	−0.0010 (6)

### (E)-4-{[(4-Methoxynaphthalen-1-yl)methylidene]amino}phenol Geometric parameters (Å, º)

**Table:**

O1—C4	1.3583 (18)	C7—C8	1.364 (2)
O1—C11	1.421 (2)	C7—H7	0.9400
O2—C16	1.3578 (18)	C8—C9	1.420 (2)
O2—H1	0.91 (3)	C8—H8	0.9400
N1—C12	1.2820 (18)	C9—C10	1.417 (2)
N1—C13	1.4192 (18)	C11—H11C	0.9700
C1—C2	1.374 (2)	C11—H11B	0.9700
C1—C9	1.4354 (18)	C11—H11A	0.9700
C1—C12	1.4585 (19)	C12—H12	0.9400
C2—C3	1.396 (2)	C13—C18	1.391 (2)
C2—H2	0.9400	C13—C14	1.3911 (19)
C3—C4	1.370 (2)	C14—C15	1.380 (2)
C3—H3	0.9400	C14—H14	0.9400
C4—C10	1.430 (2)	C15—C16	1.391 (2)
C5—C6	1.368 (2)	C15—H15	0.9400
C5—C10	1.416 (2)	C16—C17	1.391 (2)
C5—H5	0.9400	C17—C18	1.380 (2)
C6—C7	1.397 (2)	C17—H17	0.9400
C6—H6	0.9400	C18—H18	0.9400
			
C4—O1—C11	117.08 (12)	C9—C10—C5	119.68 (13)
C16—O2—H1	110.3 (19)	C9—C10—C4	119.23 (12)
C12—N1—C13	117.95 (12)	C5—C10—C4	121.08 (14)
C2—C1—C9	118.59 (13)	O1—C11—H11C	109.5
C2—C1—C12	119.94 (12)	O1—C11—H11B	109.5
C9—C1—C12	121.41 (12)	H11C—C11—H11B	109.5
C1—C2—C3	122.64 (13)	O1—C11—H11A	109.5
C1—C2—H2	118.7	H11C—C11—H11A	109.5
C3—C2—H2	118.7	H11B—C11—H11A	109.5
C4—C3—C2	119.82 (13)	N1—C12—C1	123.56 (13)
C4—C3—H3	120.1	N1—C12—H12	118.2
C2—C3—H3	120.1	C1—C12—H12	118.2
O1—C4—C3	124.66 (13)	C18—C13—C14	118.23 (13)
O1—C4—C10	114.96 (12)	C18—C13—N1	122.73 (12)
C3—C4—C10	120.38 (13)	C14—C13—N1	118.99 (12)
C6—C5—C10	120.26 (15)	C15—C14—C13	121.05 (13)
C6—C5—H5	119.9	C15—C14—H14	119.5
C10—C5—H5	119.9	C13—C14—H14	119.5
C5—C6—C7	120.45 (14)	C14—C15—C16	120.49 (13)
C5—C6—H6	119.8	C14—C15—H15	119.8
C7—C6—H6	119.8	C16—C15—H15	119.8
C8—C7—C6	120.54 (15)	O2—C16—C17	118.07 (13)
C8—C7—H7	119.7	O2—C16—C15	123.33 (13)
C6—C7—H7	119.7	C17—C16—C15	118.56 (13)
C7—C8—C9	121.15 (15)	C18—C17—C16	120.74 (13)
C7—C8—H8	119.4	C18—C17—H17	119.6
C9—C8—H8	119.4	C16—C17—H17	119.6
C10—C9—C8	117.92 (12)	C17—C18—C13	120.84 (13)
C10—C9—C1	119.34 (12)	C17—C18—H18	119.6
C8—C9—C1	122.72 (13)	C13—C18—H18	119.6
			
C9—C1—C2—C3	0.2 (2)	C6—C5—C10—C4	−178.70 (15)
C12—C1—C2—C3	177.47 (13)	O1—C4—C10—C9	−179.24 (12)
C1—C2—C3—C4	0.1 (2)	C3—C4—C10—C9	0.0 (2)
C11—O1—C4—C3	0.6 (2)	O1—C4—C10—C5	−0.7 (2)
C11—O1—C4—C10	179.71 (16)	C3—C4—C10—C5	178.48 (14)
C2—C3—C4—O1	178.93 (13)	C13—N1—C12—C1	−173.88 (12)
C2—C3—C4—C10	−0.2 (2)	C2—C1—C12—N1	29.7 (2)
C10—C5—C6—C7	−0.1 (3)	C9—C1—C12—N1	−153.10 (13)
C5—C6—C7—C8	0.2 (3)	C12—N1—C13—C18	43.94 (19)
C6—C7—C8—C9	0.1 (2)	C12—N1—C13—C14	−138.50 (14)
C7—C8—C9—C10	−0.4 (2)	C18—C13—C14—C15	−2.0 (2)
C7—C8—C9—C1	178.18 (14)	N1—C13—C14—C15	−179.68 (12)
C2—C1—C9—C10	−0.44 (19)	C13—C14—C15—C16	3.4 (2)
C12—C1—C9—C10	−177.64 (12)	C14—C15—C16—O2	175.76 (14)
C2—C1—C9—C8	−178.98 (13)	C14—C15—C16—C17	−1.9 (2)
C12—C1—C9—C8	3.8 (2)	O2—C16—C17—C18	−178.60 (13)
C8—C9—C10—C5	0.42 (19)	C15—C16—C17—C18	−0.8 (2)
C1—C9—C10—C5	−178.19 (13)	C16—C17—C18—C13	2.1 (2)
C8—C9—C10—C4	178.96 (12)	C14—C13—C18—C17	−0.7 (2)
C1—C9—C10—C4	0.35 (19)	N1—C13—C18—C17	176.85 (13)
C6—C5—C10—C9	−0.2 (2)		

### (E)-4-{[(4-Methoxynaphthalen-1-yl)methylidene]amino}phenol Hydrogen-bond geometry (Å, º)

*Cg*1 and *Cg*2 are the centroids of the naphthalene ring system (C1–C10) and the phenol ring (C13–C18), respectively.

**Table:**

*D*—H···*A*	*D*—H	H···*A*	*D*···*A*	*D*—H···*A*
O2—H1···N1^i^	0.91 (3)	1.97 (3)	2.849 (2)	160 (3)
C11—H11*A*···O2^ii^	0.97	2.53	3.409 (2)	150
C14—H14···*Cg*2^iii^	0.94	2.90	3.627 (2)	135
C18—H18···*Cg*1^iv^	0.94	2.82	3.745 (2)	170

Symmetry codes: (i) *x*, −*y*+1/2, *z*+1/2; (ii) *x*, *y*, *z*−1; (iii) *x*+1/2, −*y*+1/2, −*z*+1; (iv) *x*−1/2, *y*, −*z*+1/2.
